# From farm to table: exploring food handling and hygiene practices of meat and milk value chain actors in Ethiopia

**DOI:** 10.1186/s12889-023-15824-3

**Published:** 2023-05-16

**Authors:** Ndungu S. Nyokabi, Lisette Phelan, Gizachew Gemechu, Stefan Berg, Johanna F. Lindahl, Adane Mihret, James L. N. Wood, Henrietta L. Moore

**Affiliations:** 1 Institute for Global Prosperity, University College London, London, UK; 2 School of Geography, University of Leeds, Leeds, UK; 3 Bernhard Nocht Institute for Tropical Medicine, Hamburg, Germany; 4 Armauer Hansen Research Institute (AHRI), Addis Ababa, Ethiopia; 5 International Livestock Research Institute (ILRI), P.O. Box 30709, Nairobi, 00100 Kenya; 6 Department of Medical Biochemistry and Microbiology, Uppsala University, P.O. Box 582, 75123 Uppsala, Sweden; 7 Department of Clinical Sciences, Swedish University of Agricultural Sciences, P.O. Box 7054, 75007 Uppsala, Sweden; 8 University of Cambridge, Cambridge, UK

**Keywords:** Participatory research, Food safety, Food security, Zoonoses, One health, Visual methods

## Abstract

Livestock value chains constitute a source of livelihood for meat and milk value chain actors in Ethiopia, from dairy farmers to other associated value chain actors such as milk traders, abattoir workers, public health officials, veterinarians, butcheries selling meats, milk cooperatives, artisanal milk processors, and transporters. The development of these livestock value chains, however, is constrained by poor food safety and quality, while consumers are also exposed to public health risks due to milk and meat value chain actors’ food handling and hygiene practices.

This study used Photovoice and participant observation to explore meat and milk value chain actors’ food handling and hygiene practices in suburban areas of Addis Ababa and neighbouring Oromia in Ethiopia. The results of this study reveal that milk and meat value chain actors’ food handling practices are not aligned with the recommended Ethiopian food safety and quality standards. Low compliance with food safety and quality standards reflected a combination of factors such as lack of incentives, poor road infrastructure and low enforcement of food safety standards.

Participatory and visual research methods enable a researcher to collect context-aware data that can lead to the development of policies and intervention strategies that reflect local needs and priorities. The results of this study affirm the need to identify socially acceptable and economically viable policies and intervention strategies that are acceptable to all chain actors; and suggest there is an imperative to train milk and meat value chain actors on good hygiene handling practices, improve road infrastructure, and facilitate access equipment such as fridges and freezers that can contribute to maintaining food safety and quality.

## Introduction

Livestock value chains support the livelihoods of meat and milk value chain actors who engage in the production and trade of livestock and livestock products [1]. Animal source foods (ASF) are important sources of micro and macronutrients necessary for human growth, physical activity and cognitive function [1]. However, ASF can also constitute a vehicle for foodborne diseases, particularly if products are consumed without heat treatment and/or are uncooked [2, 3]. Milk and meat products may be contaminated by unhygienic handling, environments and/or infected animals [4, 5]. Raw milk may be contaminated with pathogens present in the farm environment—such as *Salmonella spp.*, *Escherichia coli* strains (including *E. coli* O157:H7), *Listeria monocytogenes*, and *Staphylococcus aureus* – or pathogens associated with infected animals, such as *S. aureus* (mastitis) and *Mycobacterium tuberculosis* (TB) [2]. Pathogens can also enter milk and dairy products through unhygienic handling practices, environmental contamination from utensils, contact surfaces, floors, and packaging materials, and contaminated ingredients, such as contaminated brine or starter cultures, i.e., yoghurt starter cultures [4, 6].

Transmission of foodborne diseases associated with consumption of ASF can be reduced through improvements in the hygiene of food handling and processing environments; observance of hand, equipment and utensil hygiene practices; cold storage of meals prepared in advance; observance of correct cooking temperatures; and use of good quality of water in food handling, processing and preparation [5, 7, 8]. It is therefore important that value chain stakeholders implement proper hygiene and food safety controls throughout the food value chain—“from farm to fork”—to reduce the risk of food contamination; consumers’ exposure to foodborne diseases; and the adverse economic impact of poor food safety and quality [5, 7, 8]. Compliance with food safety standards in agri-food chains is critical to realising food safety and quality [5].

Previous studies conducted in East Africa to assess meat and dairy value chain actors’ observance of good practices indicate that sanitation, temperature control, infrastructure and equipment, and personal hygiene practices, are often absent or insufficiently applied [4, 6]. Meat and dairy and value chains do not employ qualified personnel and awareness among food business operators regarding food standards is low, while key infrastructure (e.g., roads, collection centres) is not conducive to ensuring that ASF products are safe and of high quality. At governmental level, there is a lack of structures to ensure compliance with food safety standards, such as appropriate legislations, government food inspection organisations that can investigate value chain actors, laboratory capacity to perform monitoring and verification of the actual microbiological status of food products put on the market, and lack of resources along the chain with food business operators or governments [4, 9, 10].

This study focuses on Ethiopia, recognising that milk and meat value chains play an important role in the economy, and provide food, employment and livelihood opportunities for the population [11–14]. Several studies have investigated microbiological and hygiene practices in Ethiopia; these studies, however, have primarily been researcher-led and have focused on assessing the microbial quality of food and/or related drivers of food safety and quality [11, 13–20]. To the best of our knowledge, the food handling and hygiene practices of meat and milk value chain actors in Ethiopia has not been explored through participatory stakeholder-led studies. This is noteworthy given that the success of policies and intervention strategies aimed at enhancing food safety and quality hinges on understanding value chain actors’ perceptions of the challenges to realising food safety and quality and incorporating their voices in the narratives and discourses shaping policy and intervention [9, 10].

This paper examines the food handling and hygiene practices of milk and meat value chain actors in Addis Ababa city and its surrounding areas in Ethiopia using a participatory visual research method known as Photovoice, complemented by participant observation of hygiene and food handling practices, as well as facilities. This research approach enables us to engage value chain actors in exploring the challenges faced in ensuring food safety and quality and in the collection of data that can underpin the design of solutions that contribute to improving the safety and quality of ASF products produced, traded and consumed in Ethiopia. Moreover, it allows us to contribute to the literature by addressing the current paucity of participant-led research related to food safety and quality.

## Methodology

### Study area

This research was conducted as part of the Ethiopia Control of Bovine Tuberculosis Strategies (ETHICOBOTS) project which aimed to build a scientific base for exploring control of bovine tuberculosis (bTB) in dairy systems in Ethiopia. The research was undertaken in Kaliti, a sub-city of Addis Ababa, and in Holeta in the Oromia region of Ethiopia, between April and May 2021. The study areas were selected due to a number of reasons. In addition to being part of the study area for the ETHICOBOTS project, the areas were recognised as being important centres of production for milk traded and consumed in the urban areas of Addis Ababa, the capital city of Ethiopia [13, 21]. The areas also represented the dominant dairy farming systems and were deemed reflective of milk and meat quality challenges faced by actors participating in the milk and meat value chains across Ethiopia [21–23]. Finally, the areas provided an interesting context reflective of the rapid urbanisation occurring in Ethiopia which has led to increased demand for ASF, providing livelihood opportunities for dairy farmers and value chain actors, but has also leading to food safety and quality challenges in milk and meat value chains in Ethiopia [13, 21, 23]. The study area is particularly relevant to lots of other low- and middle-income countries that are undergoing rapid development and experiencing changes in their food production systems.

### Research approach

The research approach adopted in this study involved the use of Photovoice and Participant observation for data collection. Photovoice is a community-based, participatory action research (PAR) method, developed by Wang and Burris [24], that places cameras in the hands of individuals and communities to enable them to capture and tell their stories. Photovoice democratises knowledge production; promotes social justice; and empowers communities to lead the research process and participate in the development of policies that are context-aware and socially-acceptable [25]. Photovoice recognises and challenges elitist and technocratic approaches to science, driven by outside “experts”, that have increasingly failed individuals and communities, particularly concerning policy and service structuring [25]. Photovoice can drive social transformation as it produces knowledge that reflects community realities, needs, and expertise [25, 26].

Participant observation is a participatory method that can help gain insight into farmers’ and value chains actors’ food handling behaviour [27, 28]. Participant observation were based on literature review of issues related to food handling behaviour, animal health, personal hygiene and compliance with food safety and quality regulations [4, 6, 27, 28]. Poor food handling practices and non-compliance with food safety and quality regulations are known to compromise food quality at farms level and in value chains [4, 6, 11–13, 21, 27, 28].

### Photovoice process

This study employed a modified Photovoice approach to data collection, as described by Bennett and Dearden [29]. In line with the study objectives and scope, research participants were asked to take photographs that captured: (a) food safety risks including poor hygiene practise; (b) hygiene measures employed to ensure food safety and quality; (c) challenges faced that constrained their ability to implement good hygiene practices in their day-to-day activities.

Participants were selected through purposive sampling approach. The inclusion criteria for this study included: (i) a willingness to attend research-related meetings; (ii) minimum of two years’ experience undertaking meat and/or dairy value chain activities; (iii) participate in the Photovoice exercise and a training session related to the use of digital cameras, and (iv) willingness to participate in the final debrief and discussions related to photographs. During the participant recruitment process, we actively looked to identify a sample of value chain actors that was representative in terms of the gender, socioeconomic groups, and rural and urban actors in the study area.

In total, 60 individuals participated in this study (Table 1)—30 dairy farmers and 30 other milk and meat value chain actors in Kaliti and Holeta, including milk traders, abattoir workers, public health officials, veterinarians, butcheries selling meats, milk cooperatives, artisanal milk processors, and transporters. Research participants were selected through a purposive and snowball sampling approach and with the help of local experts. Each participant received a digital camera that they were asked to use for one week to document food safety risks, hygiene measures and challenges faced in their day-to-day activities. AGFA DC5500® digital cameras were provided as they were cheap; easy to use; and the battery was known to last for several days with a single charge. The research team was in regular contact with the participants via phone to offer any help with technical issues and/or use of the cameras. Participants were compensated for the equivalent of three days’ work pay for time lost associated with participation in the study (rather than engaging in their income-generating economic activities).

**Table 1:** Summary of study Photovoice participants and participant observations

	Value chain role/activity	Gender	n
Kaliti farmers photovoice	dairy farmers	Male	8
		Female	7
Holeta farmers photovoice	dairy farmers	Male	9
		Female	6
Kaliti value chain photovoice	Butcher (meat retailer)	Male	1
	Public health	Male	2
	Farmer cooperative	Female	1
	Milk trader/retailer	Female	1
	Slaughterhouse worker	Male	2
	Livestock traders	1 male/1 female	2
	Transporters	Male	2
	Artisanal processors	1 male/1 female	2
	Veterinarian	Male	2
Holeta value chain photovoice	Butcher (meat retailer)	Male	2
	Public health	Male	1
	Farmer cooperative	Female	1
	Milk trader/retailer	Female	2
	Slaughterhouse worker	Male	2
	Livestock traders	2 male/1 female	3
	Transporters	Male	2
	Artisanal processors	Female	1
	Veterinarian	Male	2
Participant observation sites/places		Number of sites/places visited	
Butchery and eateries		4	
Livestock markets		5	
Milk bulking centres/areas		2	
Milk bars/ retailers		5	
Livestock feedlots		2	
Export abattoir		1	
Domestic abattoir		1	
Milk processing company		1	
Milk cooperative		1	
Smallholder dairy farms		10	

After one week, the cameras were collected and photographs downloaded to the researcher’s laptop for printing. Participants took over 3,000 pictures; however, only 500 were deemed to be of sufficiently good quality for printing and use to guide the follow-up Photovoice discussions (i.e., in-depth, semi-structured group discussion sessions). Photographs taken by research participants were categorised to highlight food safety and handling themes. Dairy farmers arranged the photographs that they had taken into the following categories: livestock health, milking activities, milk storage, selling activities, containers and equipment used for milking and storage, and cleaning of containers used to store milk. Meat and milk value chain actors similarly assigned photographs into categories, namely, food preparation, hygiene measures, quality control, personal hygiene measure among others.

The follow-up discussions were held to understand participants’ motivation for taking each photograph and the message they wanted to communicate with the photograph. After a given participant had explained his/her photograph, other participants were invited to offer their views and comments regarding the message communicated by the photograph. The discussions were conducted in local languages, Amharic in Kaliti sub-city and Afaan-Oromo in Holeta and were recorded using a dictaphone, with participants’ consent.

Due to the COVID-19 pandemic and civil unrest in Ethiopia, it was not possible to organise a community exhibition, in line with the guidelines of Wang and Burris [24] for the Photovoice process, due to a ban on large group meetings and a travel ban that affected the research team. We were, thus, unable to produce photo books and engage the community and policymakers through photo exhibitions and follow-up Photovoice discussions.

### Participant observation process

Participant observation was undertaken by the primary author on farms and across formal and informal meat and milk value chain nodes in Addis Ababa city (Bole, Ketema and Kaliti sub-cities) and the Oromia region (Kaliti and Sendafa towns) (Table 1). Observations were based on purposive sampling of actors based on their willingness allow us conduct the observation exercise. Observation related to food handling hygiene, use of personal protective clothing (PPE), the hygiene of the food handling and/or selling environment; the cleanliness of containers and equipment; the presence of toilets and water; and the quality of infrastructure for milk and meat transport and storage. Observations were documented in the form of field notes and photographs. Consent was obtained before human subjects, or their work premises were photographed.

### Data management and analysis

The recorded discussions were transcribed verbatim and translated to English by a trained research assistant with a good command of both local languages. Translations were checked against the original transcripts to ensure accuracy. Thematic content analysis of the transcripts was undertaken using NVivo software® and followed the grounded approach process described by Bennett and Dearden [29] and Green et al. [30]. Data were coded around the themes of food safety and handling in milk and meat value chains quotes identified that provided insights into food handling and safety practices adopted by dairy farmers and value chain actors. The information collected through the Photovoice was triangulated and checked for accuracy against the field notes and photographs taken during participant observations.

Participant observations notes and photographs were used to check whether farmers and value chain actors’ practices compiled with hygienic food handling recommendations. Photographs were sorted into categories, with food safety and handling themes similar to those identified by dairy farmers and value chain actors during the Photovoice discussions.

### Ethical approval

This research had ethical clearance from the University College London Research Ethics Committee (UCL-REC) approval number 19867/001 and the Armauer Hansen Research Institute (AHRI) and ALERT hospital AHRI/ALERT Ethics Review Committee (AAERC) approval (Protocol number PO-(46/14). Written informed consent was obtained from dairy farmers and meat and milk value chain actors who were briefed, in the presence of a witness (local expert), that their participation in the study was voluntary, and that confidentiality would be maintained at all times.

## Results

**Fig. 1:**
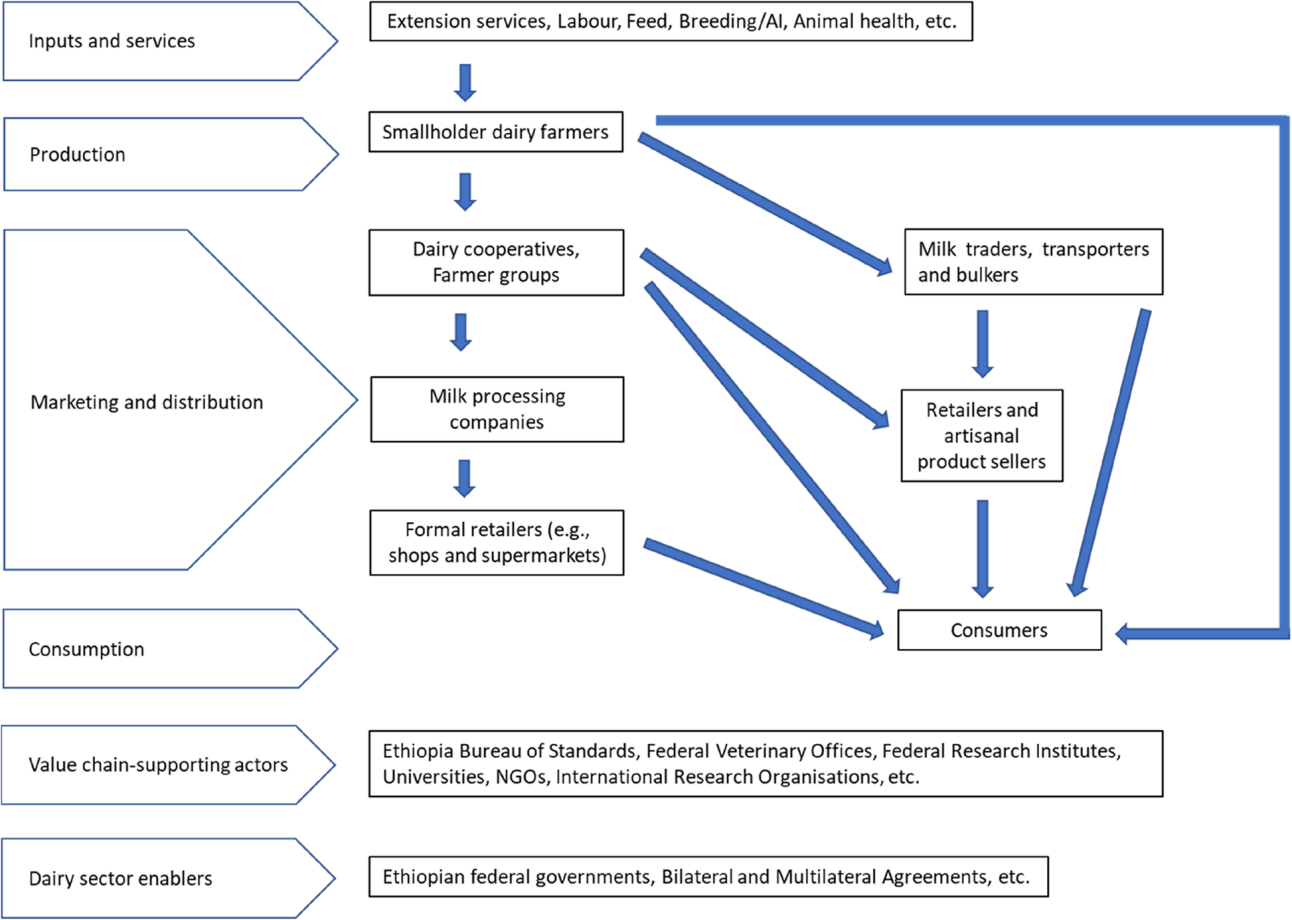
Milk value chain in Addis Ababa and surrounding Oromia region

**Fig. 2:**
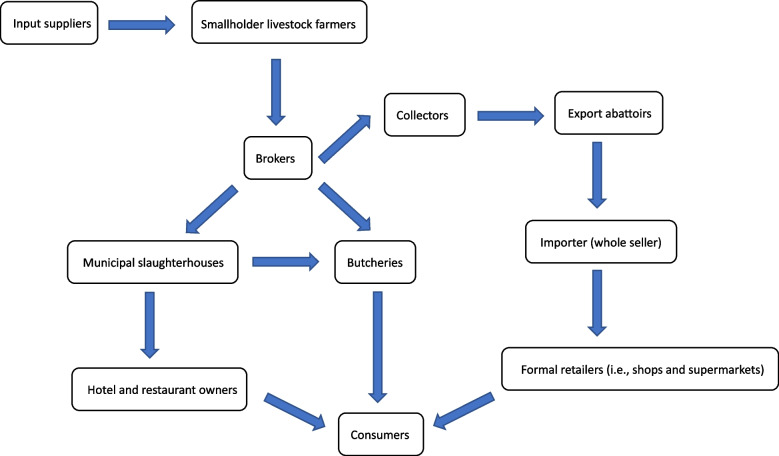
Meat value chain in Addis Ababa and surrounding Oromia region

Figure 1 presents an overview of the diverse set of actors involved in the Ethiopian milk value chain. Additionally, it indicates which actor undertakes which activity or set of activities from dairy farmers to transporters, traders, artisanal cheese and butter makers, dairy farmer groups and cooperatives, milk bars, processors, eateries, public health agents. Figure 2 presents the actors involved in the Ethiopian meat value chain who undertake various activities, from farmers to transporters, traders, slaughterhouse workers, butcheries and eateries, and public health agents. Livestock trade occurs throughout the country with animals produced in rural areas sold for export or consumption in urban markets. Value chain actors working in and around Addis Ababa reported that cattle were mainly sourced from central Ethiopia; camels were sourced from the Northern Showa, Afar and Borana regions; and sheep and goats were sourced from the Somali and Harari regions. Livestock trade followed seasonal patterns. Livestock were slaughtered for meat in either local or export slaughterhouses that served local or export markets, respectively. The majority of actors in both the meat and milk value chains had basic primary school education and were trained on food handling and safety particularly in the informal value chain by NGOs and government public inspectors who provided advice and instructions to value chain actors during premises and other inspections.

### Food safety measures compliance, food handling and hygiene practices

#### Dairy farmers’ food safety measures compliance, food handling and hygiene practices

The smallholder dairy farmers who participated in this study, either in the Photovoice exercise or the participant observation exercise, regarded milk production as an important source of income and livelihood security. Recognising that producing high-quality food products hinged on good hygienic handling and animal health practices, they reported facing several challenges in ensuring the quality of dairy and meat products, particularly those destined for the local market.

The majority of dairy farmers sold their milk immediately after milking to consumers in their locality, particularly in urban areas. Only a handful of farmers had access to a refrigerator where they could chill milk before selling it to traders in the morning. Farmers used cold water baths to maintain milk quality, particularly the quality of evening milk, and sold milk in its raw and unpasteurised form to consumers.

“Since there is not much [milk] produce in our area. [it] will be sold immediately”

Dairy farmer, Kaliti Photovoice discussion (May 2021).

“We put the container with the milk in another container with cold water and place them on the cold floor”Dairy farmer, Kaliti Photovoice discussion (May 2021)

Alluding to the fact that the majority of Ethiopians were practising Orthodox Christians, who obeyed regular fasting days when they did not consume animal products, farmers explained that milk produced on fasting days or during fasting season (lent season) was processed into butter and cheese as a value-added activity to avoid milk spoilage and post-harvest losses:

“*We make cheese in fasting times. At other times the raw milk will be sold out and there will be no leftovers to be processed into cheese* [..] *similarly we will make butter during fasting times. In other times the raw milk will be sold out immediately*.”

Dairy farmer, Kaliti Photovoice discussion (May 2021)

Cheese and butter were also prepared with unpasteurised raw milk. There was no quality-based milk payment system in the study area and dairy farmers regarded milk and dairy product prices as low, noting that both formal and informal value chains paid similar prices:

“*When merchants [informal traders] buy from us at 15 birrs, unions [cooperatives and farmer groups] also buy from us at 15 birrs. Even if we are under the organizations [cooperatives and farmer groups], we are not getting any benefit from it*.”

Dairy farmer, Kaliti farm Photovoice discussion (May 2021)

“*We are just struggling even to manage our own lives let alone feeding them. It is 18 to 19 birrs for a litre of milk*.”Holeta farm Photovoice discussion (May 2021)

Dairy farmers were of the opinion that dairy cooperatives and farmer groups could help farmers access milk markets in urban areas by coordinating milk collection and marketing on their behalf:

“*Organisation and responsible bodies in the district could find and form market linkage works. There are many huge factories in Addis Ababa which need milk. We hear that today the price of milk reaches 35 birrs. But even if I want to sell it there, I can’t do it individually while only selling 50/60 litres.* [But] *if we get organized and if a market tie between us is formed, I think our problem could be solved*.” Kaliti farm Photovoice discussion (May 2021)

#### Food safety measures compliance at value chains level

##### Food safety compliance in the formal milk value chain

Government agencies were reported by dairy farmers and milk value chain actors as demanding strict compliance with certification, including business permits and public health certificates, and actors indicated that they recognised that, for these agencies, it was a means of generating taxes at national and federal government levels. Formal value chain actors complied to a greater extent than informal value chain actors with the Ethiopian Standards Agency (ESA) food safety and quality standards and regulations, such as milk testing. Moreover, they maintained the hygiene of their premises such as having a hand washing station, clean floors, use of recommended utensils (i.e. aluminium instead of non-food grade plastic containers) as they expected regular inspection to be conducted randomly by the mandated by government agencies.

Participant observation in the formal milk value chain indicated high use of PPE by milk processing plants workers, for example, in response to regular inspections and strict enforcement of PPE use by the factory’s management staff; quality management team; and government agencies. Processors sold pasteurised packaged milk, either as short-life, pasteurised milk or long-term ultra-heat-treated milk.

##### Food safety compliance in domestic and export meat value chains

In the meat value chains, the majority of slaughterhouse workers also used PPE due to strict enforcement by the premises management and government agencies responsible for public health. However, participant observation revealed that, in some instances, PPE was not worn properly by slaughterhouse workers, notably those serving the local market. Both local and export slaughterhouses had facilities for workers to change into PPE before commencing their work shifts. Export slaughterhouses had well-organised large cubicles with lockers for workers; this was not the case in the local slaughterhouse where changing rooms were small. Moreover, export slaughterhouses had on-site laundry facilities where PPE used by workers could be washed before being reused.

“*Workers have separate rooms for changing their clothes into* [PPE] *working clothes before entering to slaughtering room”*

Public health officer, Kaliti Photovoice value chain discussion (May 2021)

Similar to milk and dairy products, trade and consumption of meat in the study area was heavily influenced by religion. The number of livestock slaughtered, for example, reflected the fasting practices of the predominant Orthodox Christian community. On fasting days and during the 40-day Lenten fasting season, meat was not sold in Orthodox butcheries as the community abstained from consumption of meat and livestock products. In slaughterhouses that served the local market, there were separate slaughter facilities for cattle, camels and shoats (sheep and goats). These facilities additionally had separate Muslim and Orthodox Christian sections to cater for the religious requirements of these communities. The export market catered mainly for Muslim halal requirements as the meat was destined primarily for purchase by consumers in the Middle East.

“*These are slaughtering sections for Muslims and Christians. We have priests who come to do prayer, bless the cattle, and sprinkle the* [slaughtering] *room with holy water before slaughtering starts. The same applies to the Muslim section as well*”

Public health officer, Kaliti Photovoice value chain discussion (May 2021)

Slaughterhouses that served the export and local market both had livestock isolation facilities for ante-mortem examination of live animals before they were slaughtered. Livestock for local consumption were observed overnight before slaughtering. Sheep and goats (shoats) destined for export were kept for several days in isolation and quarantine holding areas, including overnight in special observation pens before slaughtering. In both local and export slaughterhouses, public health officers were responsible for performing the ante-mortem examinations, as well as the post-mortem examinations of carcasses that ensured meat was in line with food safety standards before it was sold to consumers.

*“In ante-mortem, we look for many things initially when they get into the abattoir. We make them stay for 24 hours before slaughter and we will not allow any slaughter if we see anything unhealthy. After slaughtering we also look for conditions like bTB and Fasciola on internal organs* […] *it is during antemortem that good emphasis should be given. If we suspect a problem, we will isolate and make the cattle wait for the specified period*.

Public health officer, Kaliti Photovoice value chain discussion (May 2021)

**Fig. 3:**
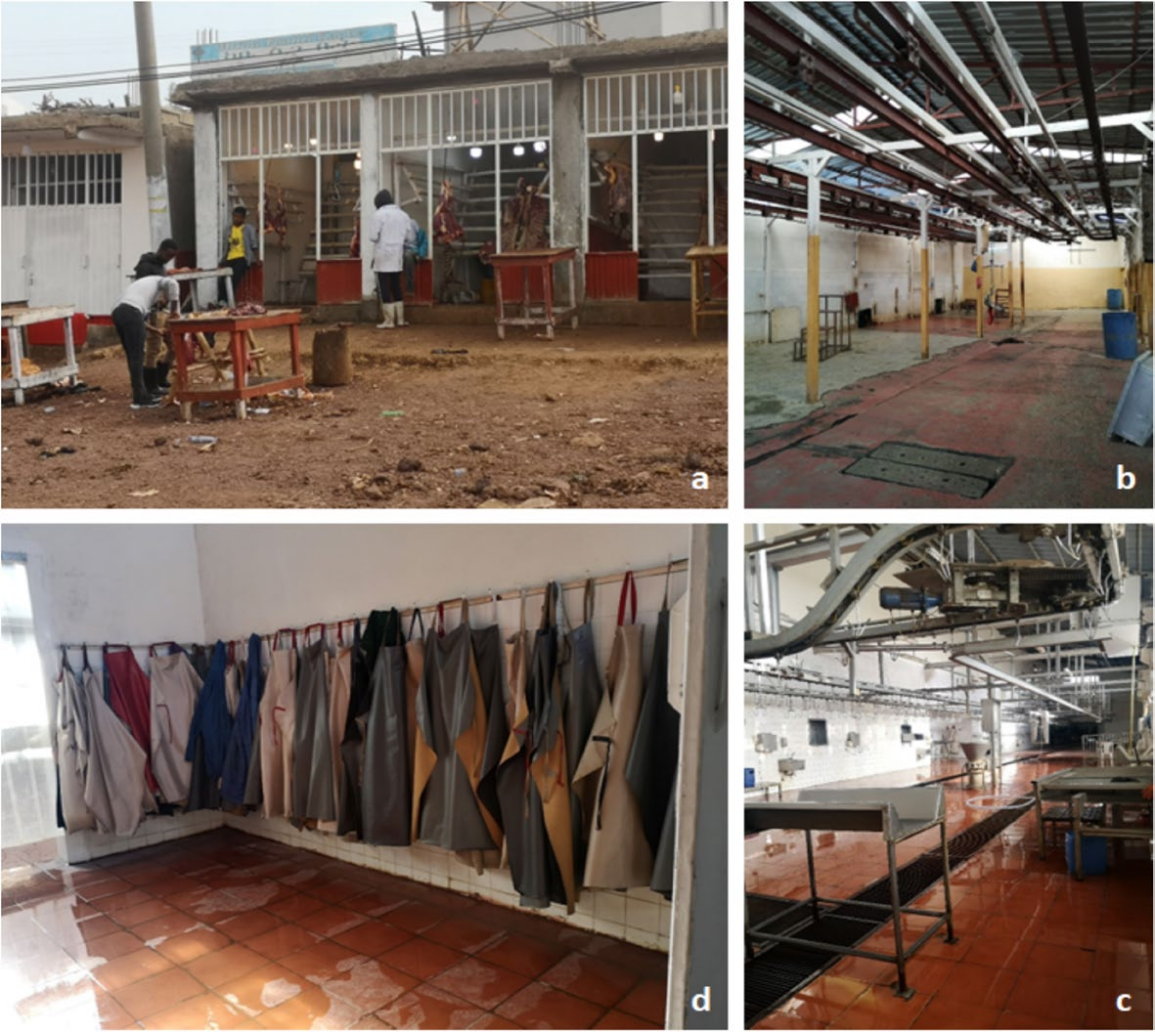
Meat value chain hygiene practices- (clockwise: (**a**) Meat butchery (**b**) Local cattle slaughterhouse (**c**) Changing room of an export slaughterhouse (**d**) Export slaughterhouse)

Figure 3 presents several photographs taken by actors in the meat value chain. Workers had access to a foot bath at the entrance of the export slaughterhouses, a disinfection chamber with a plastic screen (*a hanging plastic sheet separating two rooms*), and a mandatory hand cleaning area with hand dryers; these facilities were all absent in the local slaughterhouse. In both the export and local slaughterhouses there was continuous cleaning of the floors with pressurised water to remove blood and other waste materials. The export slaughterhouses were clean and well-maintained, i.e., the floors were intact and had no holes or cracks, compared to the local slaughterhouses where the floors had some cracks in the floor that could retain water.

“*We use pressurized water. It is a separate water tanker which is not connected with the municipality water line. So, we don’t have any water shortages. Sometimes we have electric blackouts* […] but *we do have a generator”*

Public health officer, Kaliti Photovoice value chain discussion (May 2021)

Export and local slaughterhouses had clean aluminium surfaces where meat was placed during processing. Stainless steel hooks were used for handling meat. Knives were disinfected in a special hot water electric boiler to ensure hygiene and food safety was maintained.

“*During slaughtering* [the animals] *are stunned then hanged using a hook* [then] *the throat is cut. Then they will be slid through a conveyor system* [and other workers]* will continue the process of skinning and separating the organs”*

“*Here we have a boiler line for sterilizing knives or it could also be an axe in case we find pus or anything else they will sterilize their equipment here.* [No slaughtering can start] *No one starts their work before boiled water is ready*.”

Public health officer, Kaliti Photovoice value chain discussion (May 2021)

In the export slaughterhouses, animal carcasses were washed, sprayed with ascorbic acid and chilled immediately to ensure food hygiene, safety and quality. In the local slaughterhouse, the animal carcasses were only washed and allowed to drain before being transported immediately to butcheries. Meat destined for the export market was transported chilled in sterile packaging to meet the strict food safety standards demanded by export markets.

*“…every animal part will be hung separately. The carcass will have its code to identify its owner. Every part of the cattle will have that same code and they do have their way of placement that even helps them to identify which liver or kidney is whose. Here we have liver and lungs. We do examinations for liver and lungs and they do have their hooks*”

Public health officer, Kaliti Photovoice value chain discussion (May 2021)

Meat was visually inspected by public health officials. Carcasses that were approved for consumption were stamped and recorded before being released from both local and export slaughterhouses. Meat and offal (liver, kidneys and lungs) with lesions and cysts were condemned as not suitable for human consumption. During the Photovoice follow-up discussions held to identify the message behind photographs taken, public health inspectors explained, using the pictures that showed cysts visible in lungs and livers, that led to meat being either partially or fully condemned. They stated that it was common to see bTB lesions on infected cattle lungs, and cysts in the liver caused by *Fasciola hepatica* and numerous other helminth infections.

“*We do* [meat] *examinations mainly by visual inspection* […] *we use knife and hook to incise and inspect meat parts. This* [picture] *is a liver with an abscess*. [Many times] *we find liver flukes or hydatid cysts* [and we have to] *discarded such liver. We also diagnose kidneys separately. In the case of bruises; if a specific part is bruised badly, we do condemn partially. If organs like the liver are damaged as a whole system, we also condemn that*.”

Public health officer, Kaliti Photovoice value chain discussion (May 2021)

Meat for the local market was transported without cooling, during the day or in the early evening, in transport trucks with meat boxes of steel. Transporters loading and unloading meat wore special clothes for handling meat to maintain quality and hygiene. The meat was, however, not covered during loading, transporting and unloading which exposed it to contamination from dust and flies during handling. Additionally, there was a risk of meat contamination due to a lack of handwashing by slaughterhouse workers during and after handling the meat; this reflected lower enforcement of and adherence to food safety and quality standards.

*“This is a stamp that assures legality or that ascertains the meat is healthy after examinations are done and when it is ready for shipment*. […] *We have meat transporting vehicles for meat delivery” *Public health officer, Kaliti Photovoice value chain discussion (May 2021)

Some butchery operators tried to maintain the quality of raw meat, and prevent contamination by flies and dust, by covering it with plastic food wrap. Participant observation revealed that value chain actors in butcheries had unhygienic meat handling practices, including non-disinfection of chopping boards and knives, non-use of PPE, and handling money while at the same time cutting and handling meat.

*“If meat is not consumed in a day from the abattoir, we will put it in the refrigerator. [..] The other thing, we will wrap it up with cling film [plastic food wrap] when it comes from abattoir to prevent exposure to dust”* Butchery operator, Kaliti Photovoice discussion (May 2021)

### Food safety compliance gaps at farm level and value chains

#### Food safety compliance gaps at farm level

**Fig. 4:**
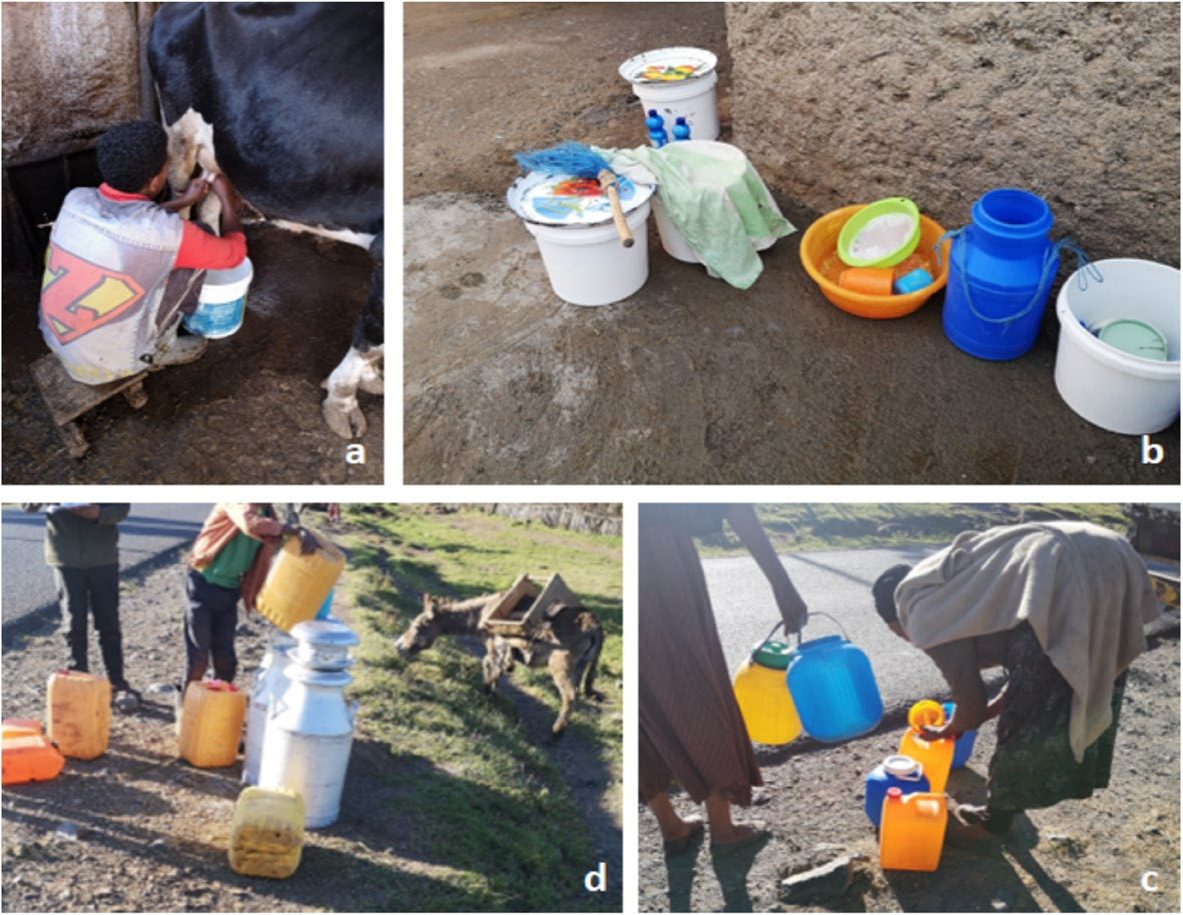
Milk handling at farm level (clockwise: (**a**) milking (**b**) milking and storage containers (**c**) Plastic milk storage and transport containers (**d**) Milk bulking by the roadside)

Photographs taken by the majority of the dairy farmers (20 out of the 30 farmers) revealed poor milking hygiene and also that cows were milked in unhygienic milking environments (see Fig. 4). Cattle shed where cattle were kept and milked were often unhygienic. Additionally, there was widespread use of non-food grade plastic containers, which were difficult to clean, for milk storage and transportation. However, nearly half of the farmers used the recommended aluminium containers and improved “*Mazzican*” (improved food-grade plastic containers that are easy to clean). Farmers indicated that they sieved milk after milking to remove contaminants such as hair and other debris.

Farmers stated, during the Photovoice follow-up discussions, that they used the same water to clean udders and teats on all cows. Participant observation confirmed that dairy farmers used the same water to clean their milking cows; this could lead to milk contamination and spread udder pathogens between cows. We observed that only a few farmers engaged in teat dipping (with a disinfecting solution) before, during and after milking; those who did so, however, recognised that it was key to ensuring milk safety and hygiene.

“*This one [photo] shows while I was drying their teats after washing it. [… if the teats are dirty] I might add detergent [and use] only boiled water (not very hot). Then [the teats] are dried with a cloth*.” Kaliti farm Photovoice discussion (May 2021)

Participant observation revealed cows with teat infections and blocked teats in five of the visited farms which could be due to unhygienic housing conditions. Farmers cited a lack of space to build spacious cattle sheds with separate living and milk areas as a constraint to ensuring a hygienic milking environment. Water used in farms was untreated and likely to be contaminated with microorganisms.

“*We clean them [the cattle] and dried their udders and teats with cloth and milk them at the same spot […] It is evident that it is not a good practice to milk them in their living area […] But that is because of the limited budget we are forced to do that after cleaning the area*.” Dairy farmer, Kaliti farm Photovoice discussion (May 2021)

Management of animal health and welfare was below the required Ethiopian food safety standards and could expose consumers to health risks. Farmers were aware that there was an imperative to improve animal health practices:

“*If I say the health of the cattle is well maintained, it would be a lie*.”

Dairy farmer, Holeta Photovoice discussion (May 2021)

#### Food safety compliance gaps at value chains level

##### Food safety compliance gaps in milk value chains

**Fig. 5:**
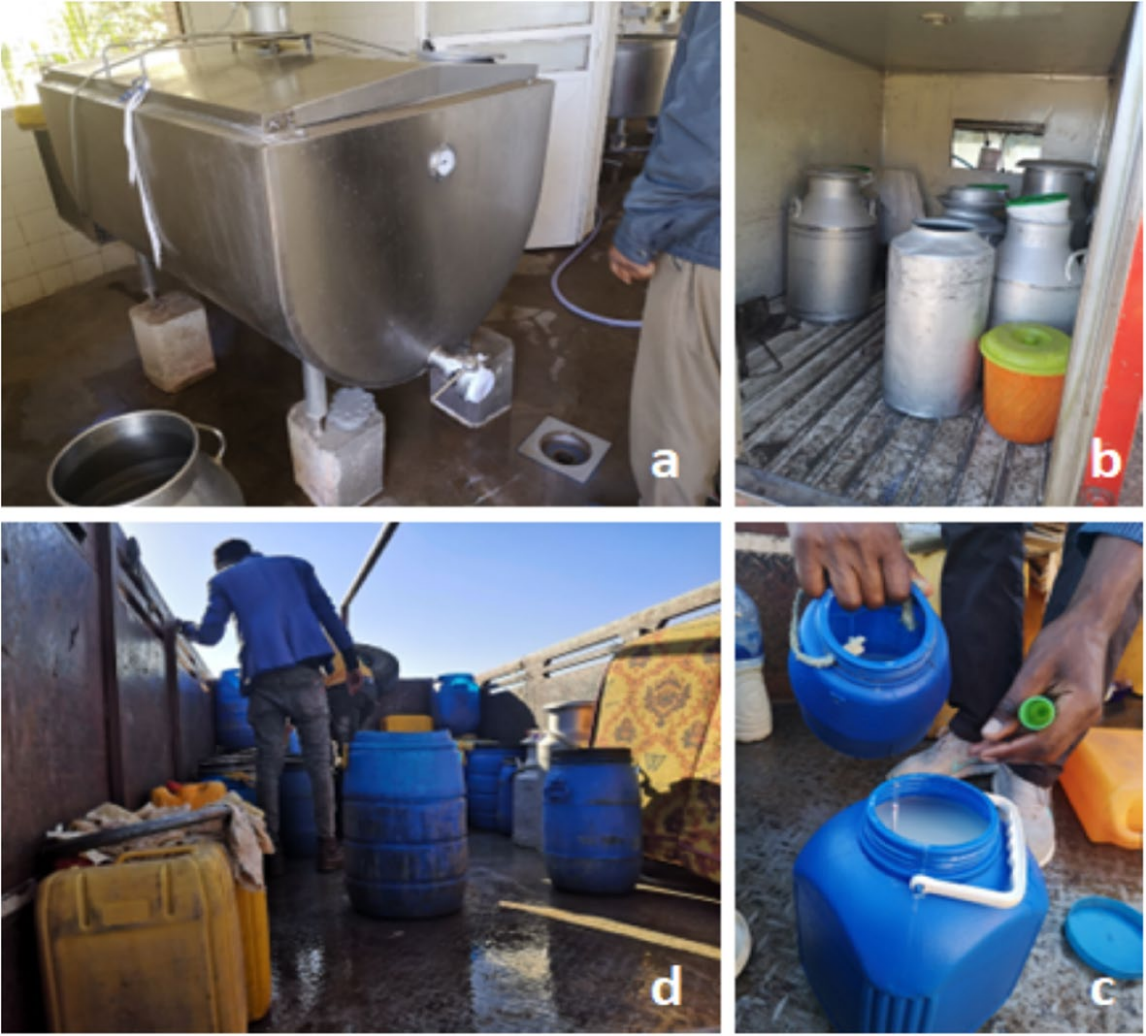
Milk handling at the dairy value chain (clockwise: (**a**) Milk bulking and storage tank in a big farm (**b**) Milking bulking and storage containers of a milk processor (**c**) Milking bulking and storage containers of an informal milk trader (**d**) Milk density testing)

There was widespread use of plastic containers for milk bulking and storage by milk value chain actors. Photovoice pictures (Fig. 5) and participant observation revealed that only a minority of actors used aluminium and improved “*Mazzican*” containers for milk transportation and storage. Containers were not clean or hygienically handled by the value chain actors; water utilised to clean equipment and containers was not treated; and no disinfectant was used in cleaning, all of which could lead to milk contamination during milk bulking, transportation and storage.

Milk bulking by transporters was undertaken in an unhygienic, open-air environment, mostly at the side of the roads and in open transport vehicles which were characterised by wet and dirty floor surfaces with no regular cleaning during the bulking process. Cooperatives, traders and transporters conducted organoleptic testing for milk quality, such as alcohol and density tests, before accepting milk for bulking. During bulking, milk was sieved to remove debris and contaminants such as cow hair. Milk was bulked and transported without cooling which could contribute to milk quality deterioration. There was low use of protective personal equipment (PPE) such as hairnets, overcoats, boots and gloves by value chain actors as required by law for people handling foods, particularly milk traders, vendors, and actors engaged in milk bulking, transportation or retailing.

The hygiene of observed bulking premises, particularly cooperatives and dairy farmer group shops, was below the recommended Ethiopian public health standards with regards to food safety and quality. The Ethiopian public health standards demand that floors be clean without cracks, have hygiene facilities such as toile and water among others. There was an absence of hygiene facilities in the milk value chain to ensure hygienic milk bulking, transport and trade. In the markets and at these collection points, there were no toilets or handwashing facilities available for value chain actors which could make it difficult to comply with hygiene requirements. Milk bulking cooling facilities were absent which could also contribute to milk quality deterioration. Moreover, the road infrastructure was mostly poor, particularly in rural areas and dairy farmers had to rely on donkeys and horses to deliver the milk to the main roads where traders with trucks bought and bulked it.

There was a lack of quality inspection by the public health department and regulatory institutions, particularly in the informal milk value chain. Milk was commonly sold to consumers raw and/or unpasteurised. Dairy products sold by artisanal actors in the informal value chains were also produced using raw and unpasteurised milk.

##### Food safety compliance gaps in meat value chains

Animal welfare was poor, with livestock transport, in particular, leading to fractures, fatigue and injuries in animals arriving at local slaughterhouses.

“*The abattoir standard is very low* [..] *in relation with animal welfare: particularly the camel section*” Public health officer, Kaliti Photovoice value chain discussion (May 2021)

In the export slaughterhouse, livestock were isolated and quarantined for some time and fed to ensure they were healthy before slaughter. Some feedlots were repurposed as quarantine facilities and for fattening of livestock destined for export markets.

Meat was hung in the open air in butcheries which could expose it to flies and dust. Few butcheries had freezers and/or fridges to keep meat overnight, while traders could only buy and trade small quantities as they could not preserve meat particularly at night and over fasting days.

Meat value chain actors were aware of contamination and disease risks related to meat consumption. In Ethiopia, there is a wide consumption of raw meat (e.g., Siga and Kitfo) and, hence, recognition that meat quality and hygiene are important public health issues. Disease outbreaks were perceived as incentivising consumers to slowly shift their meat consumption behaviours towards cooked meat dishes; meat traders reported that foodborne disease outbreaks had led to behaviour changes:

“Previously, people were looking for Kurt (raw meat) but now since there were cholera cases, they have shifted their consumption into fried meat.”

Butchery operator Kaliti Value chain Photovoice discussion (May 2021)

## Discussion

This study used a combination of Photovoice and participant observation to explore dairy farmers’ and milk and meat products value chain actors’ food handling and hygiene practices in two peri-urban study sites in central Ethiopia. The results of this study reveal that participatory visual research methods can facilitate the collection of data on food handling practices and proactive participation in research by those responsible for realising an improvement in food safety and quality from farm to glass and/or fork. Farmers and value chain actors were willing to take part in research responding to the current paucity of participant-led research regarding food safety and quality and motivated to contribute to the development of food safety-related policies and intervention strategies that are socially aware, economically viable and culturally acceptable and which can lead to sustainable food safety improvement.

### Farm-level food-handling hygiene and practices and implication for food quality and safety

The results of this study reveal low adoption of good animal health practices and biosecurity practices at farm level, which could expose cattle to diseases and zoonoses of public health concern [13, 21, 31]. Intensive dairy production in urban and peri-urban areas has increased the risk of bovine TB [32]. Extensive dairy production system, i.e., grazing in communal lands and shared watering areas, exposes cattle to diseases including zoonoses through herd mixing [31, 33].

Low use of teat dipping can lead to udder infection and high milk somatic cell counts [27, 34, 35]. Poor animal health practices are a result of dairy farmers’ lack of awareness about important diseases such as brucellosis and bTB; a lack of space to isolate and quarantine sick cattle; and poor disposal of animal tissue such as stillbirths or retained foetal material [33]. Previous studies have demonstrated that good animal health practices, such as vaccination, and adoption of biosecurity measures are crucial to guaranteeing food safety at farm level [13, 21]. Previous research in Ethiopia has documented foodborne zoonoses including bTB, *Brucella spp.* and *E. coli* [11–13, 21, 36].

Findings of this study reveal poor milking hygiene and handling and storage practices at farm level that were not in line with recommended food safety standards (Sect. 3.1). Unhygienic milking conditions; unclean milk handling equipment; and the use of contaminated water are major drives of milk microbial contamination in Ethiopia [37]. Milk microbial contamination due to food safety compliance gaps can lead to health risks for consumers [38]. The results of this study are similar to previous studies that suggest milk quality and safety are influenced by several factors at farm level, including milk handling and hygiene practices, milk storage equipment and animal health [6, 11, 39–41].

There is an imperative to create an enabling policy environment and for provision of economic incentives that encourages dairy farmers’ compliance with food safety regulations and standards [10]. Increased adoption and compliance with milk quality standards and food safety regulations could benefit smallholder farmers by increasing their market access and reducing post-harvest losses associated with rejection of milk deemed to be of poor quality [10]. It could also benefit the wider dairy sector by increasing the amount of milk intake for processing into dairy products destined for the national and export markets [42].

Milking hygiene and storage practices, as well as animal health practices, influence food safety and quality [5]. Widespread use of non-food grade plastic containers can lead to milk contamination and is contrary to regulations stipulated by Ethiopia food standards [11, 12]. Plastic containers are difficult to clean and often retain a high microbial load, even after cleaning with disinfectants [43, 44]. There is, thus, an imperative to assist dairy farmers and value chain actors in accessing recommended milking equipment and milk storage containers [10]. Dairy farmers have been shown to adopt improved specially designed plastic containers called “Mazzican” if these are cheaper and more accessible than aluminium containers [45, 46]. It is therefore important that the government provides support to farmers invest in capital intensive inputs through improved access to credit, information and tax waivers on important inputs such as aluminium containers [10].

Dairy farmers in Ethiopia typically sell raw and unpasteurised milk to their neighbours. This practice is associated with food safety risks related to milk microbial contamination, including brucellosis, *E. coli* and bTB, as documented in previous studies [11, 13, 14]. Raw milk consumption is a common practice that could expose consumers to zoonoses risks [12]. There is a need to encourage the uptake of practices, such as milk boiling, that can reduce the risks associated with microbial contamination [38]. However, milk boiling before consumption does not eliminate contaminants such as aflatoxins from mycotoxin-producing fungi, antibiotics and pesticide residues [9, 38]. This suggests that there is a need for ensuring the production of safe animal products from the farm and maintaining food safety and quality throughout the value chains.

### Milk value chain: food-handling hygiene and practices - implication for milk quality and safety

The results of this study also show low compliance by milk value chain actors with Ethiopian food safety standards. This is consistent with previous studies that have reported microbial contamination of milk and public health risks in the dairy sector [11, 13, 14]. Non- compliance with food safety standards could compromise milk safety and quality and affect the sensory quality and shelf life of processed dairy products [10]. Improving food safety and microbial quality will enhance the safety of milk produced and traded in the formal and informal value chains and is an important step to addressing food safety and security challenges in LMICs where milk plays an important role in diets [11, 14].

The safety and microbial quality of milk is determined by milk handling and hygiene practices and exposure during milking, collection, storage, distribution and consumption to unhygienic environmental conditions [45]. The widespread use of non-food grade plastic containers can lead to milk contamination and is contrary to the stipulated regulations. Milking containers and milking hygiene and storage and animal health play an important role in food safety and quality [5]. Milk contamination starts at farm level and cascades into dairy value chains in Ethiopia [37].

Milk is primarily sold raw and unpasteurised through the informal value chain in Ethiopia; if milk is not boiled before consumption, it poses a health risk to consumers. On some occasions, milk sold through the informal value chain may be boiled or pasteurised by small-scale pasteurisation units in informal markets [12, 13, 47]. The informal milk value chain is, however, popular as it tends to pay higher prices to dairy farmers; sells milk and dairy products at lower prices and in smaller quantities that suits the purchasing power of low-income consumers; and provides dairy products that meet local sociocultural expectations such as fermented raw milk products, i.e., cheese and butter [12, 13]. Milk microbial contamination risks are not exclusive to informal dairy markets but also happen in the formal value chains [9]. Nevertheless, milk sold through the formal value chain is of higher quality; pasteurised and packaged by milk processing companies; and bears the Ethiopia Bureau of Standards’ quality mark [9, 12]. There is therefore a need to ensure that more of the milk sold to consumers is pasteurised or boiled at home before consumption to eliminate the risk of foodborne diseases [11, 13, 14].

In Ethiopia, the focus of government and development agencies has been on the formalisation of the informal dairy markets through licensing and increased pasteurization concentrating on enforcement via fines, confiscation of milk, or closing off the premises of informal actors [9, 11, 13, 14]. These policies have however, not led to improved milk quality, which is similar to other East African countries [6, 9]. Consumer awareness of food safety risks and willingness to pay for improved food safety is increasing the demand for improved food handling practices that ensure milk quality and safety [11]. The results of this study show that milk value chain actors had limited access to infrastructure including clean water, electricity, sanitation, roads, cooling plants and refrigeration which makes it difficult to maintain milk quality and safety. There is a need to provide the necessary infrastructure such as treated water, roads and milk cooling plants to ensure food safety is maintained throughout the milk value chain [12, 48]. Lack of critical infrastructure, notably, sanitation facilities in markets and milk bulking areas, limits the ability of milk value chain actors to put knowledge into practice [48].

There was low use of PPE by milk value chain actors which exposed them to occupational risks, including zoonoses such as bTB and brucellosis [47]. One of the major reasons for low PPE use by milk value chain actors is a lack of knowledge about transmission routes and risks of zoonotic disease [47]. Another reason could be the cost associated with procuring PPE which may be beyond the financial resource of small traders [28, 49]. There is an imperative for training value chain actors improved knowledge and/or attitudes can underpin behaviour change and translate into improved food handling practices [48]. Moreover, there is need to assist milk value chain actors purchase PPE through improved access to credit facilities [28].

### Meat value chain: food-handling hygiene and practices - implication for food quality and safety

The results of this study reveal unhygienic meat handling practices and food safety compliance gaps in the value chains that could lead to microbial contamination. Meat safety and quality are influenced by animal health, welfare and handling hygiene [12]. Previous studies have also reported that meat value chain actors do not engage in good hygiene practices which leads to food safety risks [12]. Food contamination in meat value chains reflects poor hygiene practices during meat production, handling, storage, transportation and at the processing and packaging level [4, 5, 8]. Non-compliance with recommended hygiene practices, such as hand washing, wearing a hair covering, or maintaining cold storage, can lead to food contamination [50]. Poor water quality, poor hygiene during food preparation, unclean utensils, poor personal hygiene, and crowded and dusty shopping areas located alongside busy roads can also lead to food contamination [12, 50].

The findings of this study show that meat for export appears to be of higher quality compared to meat destined for the local markets. Previous studies have reported food safety and quality asymmetry between the domestic and export markets, particularly in East Africa, due to differences in enforcement and governance mechanisms [51]. Low food safety compliance could be linked to low access to improved infrastructure and the absence marketing arrangements that can improve food safety, such as fridges, freezers and electricity [12, 52].

In Ethiopia, beyond getting the requisite certificates, there is low compliance by meat value chain actors with food safety standards. This could be due to a perceived lack of economic incentives to improve food safety and suggests a need to provide actors with incentives to improve food handling practices [11, 12]. Actors could be compelled to improve food safety by incentives that nudge, such as price premiums, or push, such as regular inspections and sanctions [11].

Our results indicate low use of PPE among actors in the meat value chain, except by export slaughterhouse workers. Low use of PPE exposes actors to occupational risks such as exposure to zoonoses including bTB and brucellosis [47]. One of the major reasons for low PPE use in Ethiopia may be a lack of knowledge about zoonoses transmission risks [47].

Cultural practices, such as the consumption of raw meat, create conditions for the spread of foodborne diseases, including zoonoses such as bTB, brucellosis, taeniasis, echinococcosis, *E. coli*, *Salmonella spp.* from infected livestock or contaminated meat. The spread of such zoonotic diseases has been documented in Ethiopia [11, 12, 36]. A previous study by Negash et al. [53], for example, documented a high prevalence of cystic echinococcosis (49.5%) in cattle slaughtered in the Shashemane town abattoir in Ethiopia. Negash et al. [53] and Zeryehun and Alemu [36] have documented that cysts and bTB lesions can be missed during meat inspection and therefore pose a risk, particularly for consumers of raw meat.

The results of this study indicate there is a need to educate actors along the meat value chain and the wider society on food safety risks and pathogen transmission risks [6, 7]. However, given that it is difficult to incentivise behaviour practices particularly related to the consumption of raw meat, improving the safety and quality of meat in Ethiopia is key to reducing disease risks [12, 54]. Rigorous food safety testing and inspection of the hygiene practices in butcheries and eateries will increase the likelihood that meat value chain actors comply with public health regulations and food safety standards [12–14].

### Policy implication

The findings of this study reveal the public health implications of value chain actors’ poor food handling practices on public health. There is an imperative to ensure hygienic food handling, storage, preparation and compliance with public health regulations and food safety standards at the farm and value chain level to reduce microbial contamination of milk and meat produced and traded in Ethiopia [11, 54]. There is a need to go beyond enforcement and certification and ensure that milk and meat value chain actors understand the importance of improved food hygiene and handling practices [9, 12]. Provision of critical hygiene infrastructure such as infrastructure including clean treated water, electricity, sanitation, roads, cooling plants and refrigeration could enable value chain actors improve food safety throughout the value chain [6, 9, 10]. Finally, value chain actors could benefit from training tailored to their context aimed at improving their food handling and hygiene practices [7, 11]. A tailored training for value chains actors was shown to improve food safety and hygiene in milk value chain in Tanzania [6]. Improving the safety and quality of meat and milk in Ethiopia has the potential to increase demand and consumption of animal-sourced foods, which will improve dairy farmers’ profit margins and livelihoods and benefit the wider meat and dairy sectors [54, 55].

## Conclusion

Poor animal husbandry, milking hygiene and storage practices at farm level pose a threat to human health. Understanding food handling and hygiene practices of milk and meat value chain actors in Addis Ababa city and its surrounding areas in Ethiopia from the perspectives of these actors is key to developing policies and intervention strategies that are context-specific and are more likely, therefore, lead to sustainable improvements in food safety and quality. There is considerable scope and interest among those who stand to benefit the most from co-developing policies and intervention strategies in taking a bottom-up rather than top-down approach to identifying the factors that shape dairy farmers’ and milk and meat value chain actors’ behaviour; the results of this study underscore that using stakeholder-led participatory methods such as Photovoice and participatory observation facilitates the collection of credible data which can generate actionable insights for research participants, policymakers, practitioners and academics alike.

## Data Availability

The datasets generated and analysed during this study are not publicly available because of privacy concerns. Participants are potentially identifiable due to the small sample size and of the qualitative nature of much of the data. The datasets used and/or analysed during the current study are potentially available from the corresponding author on reasonable request.
